# Clinical, biochemical and genetic findings in adult patients with chronic hypophosphatasemia

**DOI:** 10.1007/s40618-021-01625-1

**Published:** 2021-07-02

**Authors:** V. Guarnieri, F. Sileri, R. Indirli, G. Guabello, M. Longhi, G. Dito, C. Verdelli, S. Corbetta

**Affiliations:** 1 Division of Medical Genetics, Fondazione IRCCS Ospedale Casa Sollievo Sofferenza, San Giovanni Rotondo, Foggia Italy; 2 Lab of Endocrine and Metabolic Research, Department of Endocrine and Metabolic Diseases, IRCCS Istituto Auxologico Italiano, Milan, Italy; 3 Department of Clinical Sciences and Community Health, University of Milan, Milan, Italy; 4 Endocrinology Unit, Fondazione IRCCS Cà Granda Ospedale Maggiore Policlinico, Milan, Italy; 5 Reumatology Unit, IRCCS Istituto Ortopedico Galeazzi, Milan, Italy; 6 Endocrinology and Diabetology Service, IRCCS Istituto Ortopedico Galeazzi, Milan, Italy; 7 Laboratory of Experimental Endocrinology, IRCCS Istituto Ortopedico Galeazzi, Milan, Italy; 8 Department of Biochemical, Surgical and Dental Sciences, University of Milan, Milan, Italy

**Keywords:** Hypophosphatasemia, Osteoporosis, Osteopenia, Fractures, Total alkaline phosphatase, *ALPL*

## Abstract

**Purpose:**

The study aimed to define the clinical, biochemical and genetic features of adult patients with osteopenia/osteoporosis and/or bone fragility and low serum alkaline phosphatase (sALP).

**Methods:**

Twenty-two patients with at least two sALP values below the reference range were retrospectively enrolled after exclusion of secondary causes. Data about clinical features, mineral and bone markers, serum pyridoxal-5’-phosphate (PLP), urine phosphoethanolamine (PEA), lumbar and femur bone densitometry, and column X-ray were collected. Peripheral blood DNA of each participant was analyzed to detect *ALPL* gene anomalies.

**Results:**

Pathogenic *ALPL* variants (p*ALPL*) occurred in 23% and benign variants in 36% of patients (b*ALPL*), while nine patients harbored wild-type alleles (wt*ALPL*). Fragility fractures and dental anomalies were more frequent in patients harboring p*ALPL* and b*ALPL* than in wt*ALPL* patients. Of note, wt*ALPL* patients comprised women treated with tamoxifen for hormone-sensitive breast cancer. Mineral and bone markers were similar in the three groups. Mean urine PEA levels were significantly higher in patients harboring p*ALPL* than those detected in patients harboring b*ALPL* and wt*ALPL*; by contrast, serum PLP levels were similar in the three groups. A 6-points score, considering clinical and biochemical features, was predictive of p*ALPL* detection [*P* = 0.060, OR 1.92 (95% CI 0.972, 3.794)], and more significantly of p*ALPL* or b*ALPL* [*P* = 0.025, OR 14.33 (95% CI 1.401, 14.605)].

**Conclusion:**

In osteopenic/osteoporotic patients, single clinical or biochemical factors did not distinguish hypophosphatasemic patients harboring p*ALPL* or b*ALPL* from those harboring wt*ALPL*. Occurrence of multiple clinical and biochemical features is predictive of *ALPL* anomalies, and, therefore, they should be carefully identified. Tamoxifen emerged as a hypophosphatasemic drug.

## Introduction

Hypophosphatasemia can be detected during the diagnostic workup in adult patients with osteopenia/osteoporosis and/or bone fragility. A number of disorders and drugs are known to determine hypophosphatasemia. Among these, it is mandatory to distinguish the genetic disease from acquired disorders for the following reasons: (1) the genetic disorder, known as hypophosphatasia (HPP), may be inherited and, therefore, genetic counseling in adult patients should be considered; (2) HPP may potentially benefit of the specific enzymatic asfotase alpha replacement therapy [1, 2]; (3) in patients with HPP, treatment with antiresorptive drugs may increase the risk of atypical fractures [3, 4], and, therefore, it should be avoided; (4) secondary causes of hypophosphatasemia need targeted therapeutic interventions.

HPP is a rare genetic disease characterized by low tissue-non-specific alkaline phosphatase (ALP) activity and hypophosphatasemia. It is caused by loss-of-function coding mutations of the tissue-nonspecific isoenzyme of alkaline phosphatase (*ALPL*) gene or large genomic deletion mapping at the corresponding locus on chr.1p36.1-p34. The HPP estimated prevalence in Europe consists of 1/300,000 in severe cases and of 1/6370 in moderate cases [5]. HPP displays a wide range of severity in its phenotype, from death in utero to asymptomatic disease accidentally diagnosed in adult life [6].

The genetic and acquired etiologies can be hardly distinguishable in adults considering that clinical as well as biochemical parameters are highly heterogeneous [6]. Adults may be essentially asymptomatic, or have nonspecific symptoms such as joint pain or loss of secondary teeth, and low bone mineral density (BMD). Joint pain and restricted motion are common in HPP as a consequence of chondrocalcinosis, pseudogout, and ostearthropathy. Some patients are disabled with recurrent poorly healing fractures [7]. Skeletal symptoms of HPP in adults include rickets, osteomalacia, osteopenia and osteoporosis; dental symptoms include premature tooth loss, periodontal disease and recurrent caries [8]. Additional organs involved in HPP patients are the following: skeletal muscle (weakness, chronic pain), kidney (nephrocalcinosis, hypercalciuria, severe damage), lung (respiratory failure), and central nervous system (seizures). Therefore, it is evident that nonspecific symptoms, shared with most common diseases, contribute to omit the HPP diagnosis, an eventuality more likely in adult patients with mild phenotypes. Mild HPP is most often caused by heterozygous *ALPL* mutations that have a dominant negative effect leading to low serum ALP levels [9]. Only a minority of heterozygous carriers of such dominant mutations seems to develop typical signs of HPP, suggesting low penetrance [9].

It has been reported that persistent hypophosphatasemia occurs in 0.18% of the adult general population [10] and in 0.49% of a clinic-based population of adult osteoporotic patients [11]. If low serum ALP is confirmed on repeat testing and other causes of low ALP are excluded, elevation of serum pyridoxal-5’-phosphate (vitamin B6/PLP) and/or elevation of urinary of phosphoethanolamine (PEA) support the diagnosis of HPP. PLP and PEA are ALP substrates accumulating in plasma and urine, respectively [12]. Only one of these biomarkers may be elevated in some patients with HPP. The finding of a mutation in the *ALPL* gene provides additional level of confirmation, though is not a requirement for the clinical diagnosis of HPP [7].

The aim of the present study was to investigate the clinical, biochemical and genetic features of persistent hypophosphatasemic patients from a cohort of adults referred for osteopenia/osteoporosis, fragility fractures and cancer treatment-induced bone loss (CITBL).

## Materials and methods

### Study design

This is a single-center cross sectional study, retrospectively enrolling 22 adults with unexplained persistent hypophosphatasemia. Patients with at least two values of serum total ALP below the reference levels referred to the Rheumatology Unit and to the Endocrinology Service of IRCCS Istituto Ortopedico Galeazzi in Milan, Italy for diagnosis and management of osteopenia/osteoporosis, cancer treatment-induced bone loss (CTIBL) and/or fragility fractures were enrolled. CTIBL was defined as bone loss associated with cytotoxic and/or hormonal treatments in patients with hormone-sensitive breast cancers [13].

Hypophosphatasemic patients with diseases and therapies known to reduce ALP levels were excluded by means of an extensive clinical and biochemical workup to identify causes of secondary osteoporosis. In particular, the following causes were excluded by means of history, clinical examination and/or biochemical testing: active cancer or multiple myeloma, cardiac bypass surgery, celiac disease, cleidocranial dysplasia, overt hypothyroidism, malnutrition, major trauma, massive transfusion, Mseleni joint disease, pernicious or profound anemia, protein deficiency, renal osteodystrophy, sepsis and/or multiorgan/hepatic failure, starvation, vitamin C deficiency, Wilson’s disease, zinc or magnesium deficiency, bisphosphonate therapy, ongoing chemotherapy, glucocorticoids, milk-alkali syndrome, radioactive heavy metals and vitamin D intoxication.

### Genetic analysis

Molecular studies carried out in this work were based on routine clinical care, thus not requiring any IRB approval, but, as established by the Italian Privacy Laws, only the approved informed consent signed by the patients; consent form was approved by the local ethical committee in Ospedale Sollievo della Sofferenza in San Giovanni Rotondo (FG), Italy.

#### Sanger sequencing

DNA was PCR amplified, and amplicons were purified (ExoSap-It, Affymetrix, USB Corporation, Cleveland, Ohio, USA) and sequenced (Big Dye Terminator Cycle Sequencing Kit v 1.1, and ABI3130XL Sequencer, Foster City, CA) for all the 12 coding exons (primer sequences and PCR cycling conditions upon request). Selected variants were interpreted according to the American College of Medical Genetics and Genomics/Association for Molecular Pathology (ACMGG/AMP) [14].

#### MLPA

Large genomic deletions were searched by the MLPA commercial kit (P484-ALPL, MRC Holland) following the manufacturer’s instructions.

### Clinical evaluation

Medical history was investigated in all the participants focusing on concomitant diseases and/or therapies known to induce low ALP levels and on the identification of the following symptoms and signs suggestive of HPP: occurrence of fragility fractures, familiarity for fragility fractures, neurologic events (seizure), joint disorders, muscle symptoms and skeletal deformities. Anthropometric parameters (body weight, height and body mass index calculation) were collected in all patients.

### Biochemical and hormonal assessment

Bone metabolism was evaluated with determination of serum total calcium, parathyroid hormone (PTH), 25hydroxyvitamin D (25OHD), creatinine clearance and urine calcium on a 24-h collection instruction. All patients underwent an extensive workup to investigate secondary causes of osteoporosis including endocrinological, rheumatologic or hematologic disorders, bowel diseases, chronic hepatic or renal diseases, alcoholism, and hypogonadism in males, according to clinical indications.

Biochemical analyses, including serum pyridoxal-5-phosphate/vitamin B6 (PLP) and urinary phosphoethanolamine (PEA), were obtained in all the participants. Serum PLP was determined by HPLC with fluorimetric detection (inter- and intra-assay coefficient of variations were 1.46 and 2.46%), while urine PEA was assayed by ion exchange chromatography (inter- and intra-assay coefficient of variations were < 10%). Assays were performed in laboratories external to IRCCS Istituto Ortopedico Galeazzi. All causes, except HPP, known to modulate PLP [15] and PEA [16] levels were excluded based on history and clinical features.

### Bone mineral density measurement

Bone mineral density (BMD) was measured at lumbar spine (LS), femoral neck (FN) and total hip (TH) by dual-energy X-ray absorptiometry (DXA, Hologic Discovery W or Lunar densitometers). In postmenopausal women and in men older than 50 years, osteopenia was defined by BMD T-score between − 1 and − 2.5, while osteoporosis was defined by BMD T-score lower than − 2.5, according to the World Health Organization criteria [17]. Dorsal and lumbar spine conventional X-ray was obtained in all the participants for the detection of morphometric vertebral fractures according the Genant’s semiquantitative method [18].

### Six-point clinical score

A six-point clinical score was developed to evaluate the prediction of multiple risk criteria of HPP for the occurrence of genetic variants in the *ALPL* gene sequence; the following items have been considered: (1) dental anomalies (recurrent caries, early loss of permanent dental elements, periodontal disease); (2) musculoskeletal symptoms (scoliosis, dorsal hyperkiphosis, joint pain, joint swelling, muscle pain, muscle cramp, tired legs, bone pain, full body pain, decreased mobility, chondrocalcinosis); (3) mineral metabolism anomalies (kidney stones, hypercalciuria defined as 24 h urine calcium excretion > 4.0 mg/kg body weight); (4) fragility fractures at any skeletal segment; (5) first degree relatives affected with fragility fractures; (6) increased levels of urine PEA and/or serum PLP.

### Statistical analysis

Data were obtained from medical records and statistical analysis was conducted using Prims 6.0. Qualitative variables were expressed as absolute and relative frequencies. A Shapiro–Wilk test was used to investigate distributions of quantitative variables. Normally distributed quantitative variables were expressed as mean ± standard error media (SEM) and compared by one way-ANOVA corrected for multiple comparisons. Predictive effect of the 6-points score for the detection of the *ALPL* variants was tested by bias-reduced logistic regression [19]. Biased-reduced logistic regression analysis was also used to test the association of the independent variables age, serum 25OHD and urine PEA levels, neck T-score, hypercalciuria/kidney stones, familiarity for fragility fractures and the occurrence of vertebral fracture. A *p*-value < 0.05 was considered statistically significant.

## Results

### Genetic variants of ALPL in the series of hypophosphatasemic patients

**Fig. 1:**
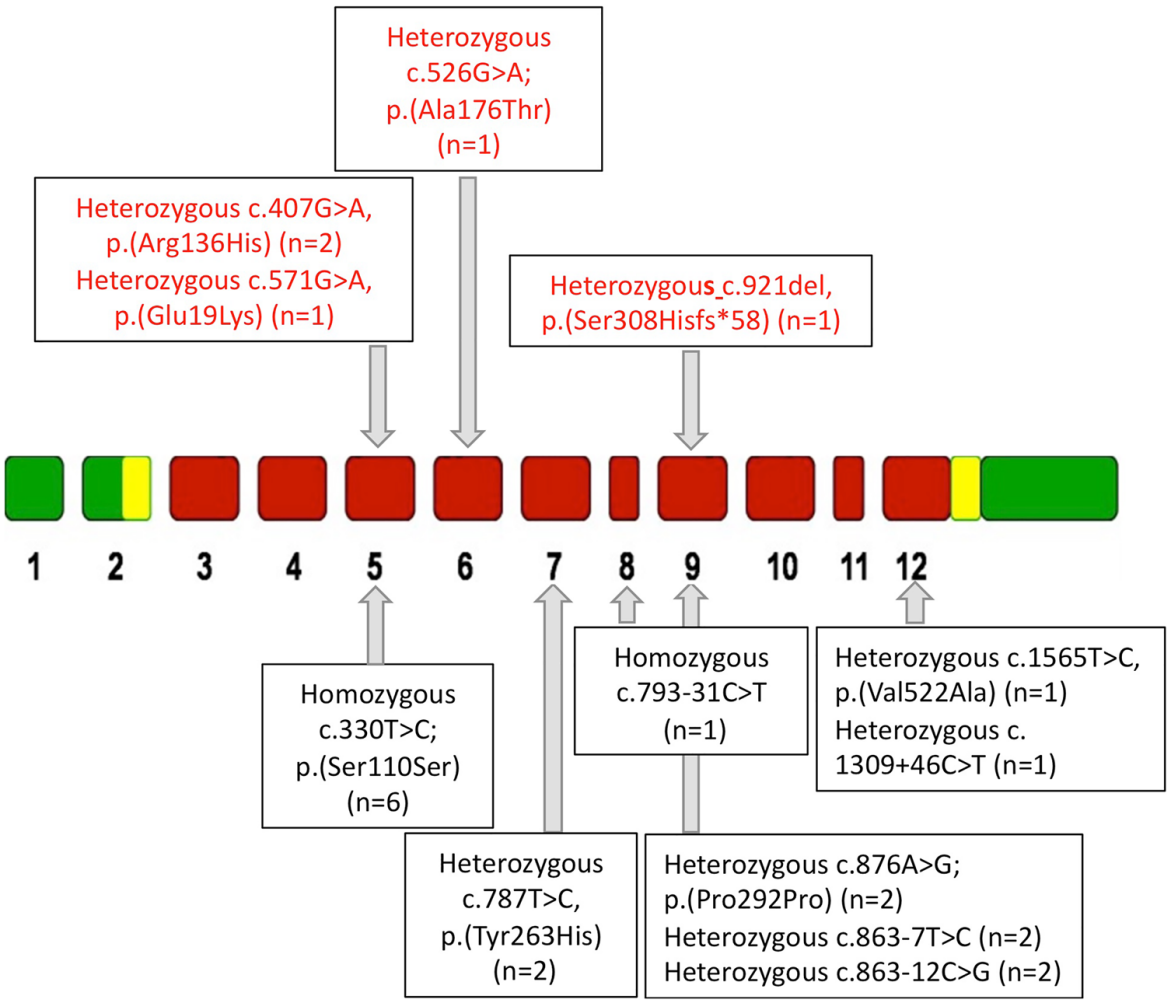
Schematic representation of the identified ALPL gene variants. Pathogenic ALPL variants were indicated in red in the upper part of the figure, while benign ALPL variants were indicated in black in lower part of the figure

Peripheral blood DNA from 22 patients (mean age ± SEM, 56.7 ± 2.4 years, 17 females, 5 males) referred for the evaluation of osteopenia/osteoporosis, fragility fractures or CITBL presenting persistent unexplained hypophosphatasemia was screened for genetic aberrations of the *ALPL* gene. Genetic analysis identified five patients (four females, one male) harboring pathogenic or likely-pathogenic *ALPL* variants (p*ALPL*) and wifhr patients harboring benign, likely benign *ALPL* variants (b*ALPL*; five females, three males) (Fig. 1). Of note, multiple *ALPL* variants were detected in seven patients. In nine patients (eight females, one male), genetic analysis failed in identifying variants in the *ALPL* gene sequence, detecting wild-type alleles (wt*ALPL*) (Table 1). Therefore, p*ALPL* variants occurred in 23% of hypophosphatasemic osteopenic/osteoporotic patients, while b*ALPL* variants occurred in 36% of patients.

**Table 1:** Genetic alterations of the *ALPL* gene detected in the analyzed osteopenic/osteoporotic patients with persistent hypophosphatasemia

Patient ID	Exon/Intron	dbSNP*	DNA	Protein	Clinical significance	Haplotype
1	Exon 9	**–**	c.921del	p.(Ser308Hisfs*58)	Pathogenic	Heterozygosity
2	Exon 6	rs121918019	c.526G > A	p.(Ala176Thr)	Likely pathogenic, pathogenic	Heterozygosity
	Exon 5	rs1780316	c.330 T > C	p.(Ser110Ser)	Likely benign, benign	Heterozygosity
	Intron 8	rs1256328	c.793-31C > T	–	Benign	Homozygosity
	Exon 12	rs34605986	c.1565 T > C	p.(Val522Ala)	Likely benign, benign	Heterozygosity
3	Exon 5	rs121918011	c.407G > A	p.(Arg136His)	Pathogenic,uncertain-significance	Heterozygosity
4	Exon 5	rs121918011	c.407G > A	p.(Arg136His)	Pathogenic,uncertain-significance	Heterozygosity
5	Exon 6	rs121918007	c.571G > A	p.(Glu191Lys)	Pathogenic-likely, pathogenic	Heterozygosity
6	Exon 5	rs1780316	c.330 T > C	p.(Ser110Ser)	Likely benign, benign	Homozygosity
	Exon 5	rs149344982	c.455G > A	p.(Arg152His)	Likely benign, benign	Heterozygosity
	Intron 3	rs1767430	c.181 + 52C > A	–	Benign	Heterozygosity
7	Exon 5	rs1780316	c.330 T > C	p.(Ser110Ser)	Likely benign, benign	Homozygosity
8	Exon 5	rs1780316	c.330 T > C	p.(Ser110Ser)	Likely benign, benign	Homozygosity
9	Exon 5	rs1780316	c.330 T > C	p.(Ser110Ser)	Likely benign, benign	Homozygosity
	Exon 7	rs3200254	c.787 T > C	p.(Tyr263His)	Benign	Heterozygosity
	Exon 9	rs3200255	c.876A > G	p.(Pro292Pro)	Benign	Heterozygosity
	Intron 9	rs74063111	c.863-7 T > C	–	Benign	Heterozygosity
	Intron 9	rs75829132	c.863-12C > G	–	Benign	Heterozygosity
	Intron 11	rs4654760	c.1309 + 46C > T	–	Benign	Heterozygosity
10	Exon 5	rs1780316	c.330 T > C	p.(Ser110Ser)	Likely benign, benign	Homozygosity
	Exon 5	rs3200254	c.787 T > C	p.(Tyr263His)	Benign	Heterozygosity
	Exon 9	rs3200255	c.876A > G	p.(Pro292Pro)	Benign	Heterozygosity
	Intron 9	rs74063111	c.863-7 T > C	–	Benign	Heterozygosity
	Intron 9	rs75829132	c.863-12C > G	–	Benign	Heterozygosity
11	Exon 5	rs1780316	c.330 T > C	p.(Ser110Ser)	Likely benign, benign	Homozygosity
	Exon 5	rs149344982	c.455G > A	p.(Arg152His)	Likely benign, benign	Heterozygosity
12	Exon 7	rs3200254	c.787 T > C	p.(Tyr263His)	Benign	Heterozygosity
	Intron 8	rs2275377	c.862 + 20G > T	–	Benign	Heterozygosity
	Intron 8	rs2275376	c. 862 + 51G > A	–	Benign	Heterozygosity
	Intron 8	rs2275375	c.862 + 58C > T	–	Benign	Heterozygosity
13	Intron 3	rs1767430	c.181 + 52C > A	–	Benign	Heterozygosity
	Exon 5	rs1780316	c.330 T > C	p.(Ser110Ser)	Likely benign, benign	Heterozygosity
	Intron 5	rs564375559	c.472 + 12insG	–	Benign	Heterozygosity
	Exon 7	rs3200254	c.787 T > C	p.(Tyr263His)	Benign	Heterozygosity
	Intron 8	rs2275377	c.862 + 20G > T	–	Benign	Heterozygosity
	Intron 8	rs2275376	c. 862 + 51G > A	–	Benign	Heterozygosity
	Intron 8	rs2275375	c.862 + 58C > T	–	Benign	Heterozygosity
	Exon 9	rs3200255	c.876A > G	p.(Pro292Pro)	Benign	Heterozygosity
	Intron 9	rs75829132	c.863-12C > G	–	Benign	Heterozygosity
	Intron 9	rs74063111	c.863-7 T > C	–	Benign	Heterozygosity

*dbSNP, single nucleotide polymorphism database

### Clinical features in hypophosphatasemic patients according the ALPL genotype

Clinical features associated with each *ALPL* variant are reported in Table 2. Of note, benign and likely-benign *ALPL* variants, some reported in the diseased as well as in general populations, were harbored by patients presenting adult HPP clinical features. Patients harboring multiple *ALPL* variants did not differ from those harboring single *ALPL* variants in terms of clinical presentation.

**Table 2:** Clinical features associated with each ALPL variant detected in the present series of adult hypophosphatasemic patients and comparison with available data in literature and/or public database

*ALPL* variants	dbSNP ID	ACMGG/AMP classification*	Patient ID	Patients clinical features	Reported clinical features
c.181 + 52C > A	rs1767430	Benign	6	Recurrent abortions, multiple vertebral fractures, hyperkyphosis, premature loss of teeth, hypercalciuria, familiarity for osteoporotic fractures	No report available
			13	Costal fracture, joint and muscular pain, familiarity for HPP	
c.330T > C, p.(Ser110Ser)	rs1780316	Benign	2	Adrenal cancer, familiarity for HPP	Found in patients with atypical femur fractures and in general population with the same frequency [27]
			6	Recurrent abortions, multiple vertebral fractures, hyperkyphosis, premature loss of teeth, hypercalciuria, familiarity for osteoporotic fractures	
			7	Pituitary macroadenoma, joint pain, familiarity for osteoporotic fractures	
			8	Multiple vertebral fractures, joint and muscular pain, premature loss of teeth, kidney stones	
			9	Multiple vertebral fractures, seizure, kidney stones, hypercalciuria	
			10	Premature loss of teeth, hypercalciuria, familiarity for HPP	
			11	Breast cancer	
			13	Rib fracture, joint and muscular pain, familiarity for HPP	
c.407G > A, p.(Arg136His)	rs121918011	Pathogenetic	3	Multiple vertebral fractures, premature loss of teeth, kidney stones, goiter	This variant was identified either as single mutation or in compound heterozigosity with other pathogenic *ALPL* variants in infantile, childhood, adult and odontoHPP (ALPLDB)
			4	Multiple vertebral fractures, recurrent caries, hyperkyphosis, joint and muscular pain, familiarity for osteoporotic fractures	
c.455G > A, p.(Arg152His)	rs149344982	Benign	6	Recurrent abortions, multiple vertebral fractures, hyperkyphosis, premature loss of teeth, hypercalciuria, familiarity for osteoporotic fractures	Fragility fractures, premature loss of teeth, joint and muscular pain (personal data by Guarnieri V.)
			11	Breast cancer	
c.472 + 7C > G	rs564375559	Benign	13	Costal fracture, joint and muscular pain, familiarity for HPP	No report available
c.526G > A, p.(Ala176Thr)	rs121918019	Pathogenetic	2	Adrenal cancer, familiarity for HPP	This variant was identified either as single mutation or in compound heterozigosity with other pathogenic *ALPL* variants in infantile, childhood, adult and odontoHPP (ALPLDB)
c.571G > A, p.(Glu191Lys)	rs121918007	Pathogenetic	5	Multiple vertebral fractures, clavicular fracture, recurrent caries	This variant was identified either as single mutation or in compound heterozigosity with other pathogenic ALPL variants in infantile, childhood, adult and odontoHPP (ALPLDB)
c.787T > C, p.(Tyr263His)	rs3200254	Benign	9	Multiple vertebral fractures, seizure, kidney stones, hypercalciuria	Found in patients with atypical femur fractures and in general population with the same frequency [27]
			10	Premature loss of teeth, hypercalciuria, familiarity for HPP	
			12	Autoimmune hypothyroidism, rib fractures, kidney stones, hypercalciuria	
			13	Costal fracture, joint and muscular pain, familiarity for HPP	
c.793-31C > T	rs1256328	Benign	2	Adrenal cancer, familiarity for HPP	Associated with kidney stones in a Taiwanese [28], and in the Han Chinese populations [29]
c.862 + 20G > T	rs2275377	Benign	12	Autoimmune hypothyroidism, rib fractures, kidney stones, hypercalciuria	Found in linkage disequilibrium in patients with mild HPP (childhood and adult HPP, odontoHPP, and perinatal benign HPP) [9]
			13	Rib fracture, joint and muscular pain, familiarity for HPP	
c.862 + 51G > A	rs2275376	Benign	12	Autoimmune hypothyroidism, rib fractures, kidney stones, hypercalciuria	Found in linkage disequilibrium in patients with mild HPP (childhood and adult HPP, odontoHPP, and perinatal benign HPP) [9]
			13	Rib fracture, joint and muscular pain, familiarity for HPP	
c.862 + 58C > T	rs2275375	Benign	12	Autoimmune hypothyroidism, rib fractures, kidney stones, hypercalciuria	Found in linkage disequilibrium in patients with mild HPP (childhood and adult HPP, odontoHPP, and perinatal benign HPP) [9]
			13	Rib fracture, joint and muscular pain, familiarity for HPP	
c.863-7T > C	rs74063111	Benign	9	Multiple vertebral fractures, seizure, kidney stones, hypercalciuria	No report available
			10	Premature loss of teeth, hypercalciuria, familiarity for HPP	
			13	Costal fracture, joint and muscular pain, familiarity for HPP	
c.863-12C > G	rs75829132	Benign	9	Multiple vertebral fractures, seizure, kidney stones, hypercalciuria	No report available
			10	Premature loss of teeth, hypercalciuria, familiarity for HPP	
			13	Costal fracture, joint and muscular pain, familiarity for HPP	
c.876A > G, p.(Pro292Pro)	rs3200255	Benign	9	Multiple vertebral fractures, seizure, kidney stones, hypercalciuria	Found in patients with atypical femur fractures and in general population with the same frequency [27]
			10	Premature loss of teeth, hypercalciuria, familiarity for HPP	
			13	Costal fracture, joint and muscular pain, familiarity for HPP	
c.921delG p.(Ser308Hisfs*58)	NR	Pathogenetic	1	Breast cancer, vertebral, wrist and humerus fractures, recurrent caries, scoliosis, familiarity for osteoporotic fractures	No report available
c.1309 + 46C > T	rs4654760	Benign	9	Multiple vertebral fractures, seizure, kidney stones, hypercalciuria	No report available
c.1565T > C, p.(Val522Ala)	rs34605986	Benign	2	Adrenal cancer, familiarity for HPP	Found in patients with atypical femur fractures and in general population with the same frequency [27]

*dbSNP* Single Nucleotide Polymorphism database (https://www.ncbi.nlm.nih.gov/snp/), *ALPL* alkaline phosphatase gene, *ALPLDB* The Tissue Nonspecific Alkaline Phosphatase Gene Mutations Database (http://alplmutationdatabase.hypophosphatasie.com/), *ID* patient identification number used in Table 1, *HPP* hypophosphatasia, *NR* Not Reported

*Variants were classified following the ACMGG Guidelines [14], as pathogenic, likely pathogenic or Variants of Uncertain Significance (VUS) with the following criteria: (1) null variant (nonsense, frameshift, deletion, insertion, canonical ± 1 or ± 2 splicing site) in genes previously described as disease-causing by haploinsufficiency/loss-of-function; (2) mutational hot spot and/or variant located in a critical functional domain; (3) variant absent in allele frequency population databases (ExAC, gnomAD, dbSNP); (4) variant reported with a minor allele frequency (MAF) significantly lower than expected; (5) variant annotated as pathogenic in ClinVar/LOVD databases; (6) co-segregation with disease in affected family members; (7) in vitro/in vivo functional studies supportive of a damaging effect on the gene/gene product. Common (MAF > 0.01) and synonymous variants were classified as benign

We compared the clinical and biochemical features among the three genotyped groups of hypophosphatasemic patients. The patients of the three groups did not differ for age and BMI (Table 3). Hypophosphatasemic patients showed similar bone demineralization, while fragility fractures were more frequently detected in patients harboring p*ALPL* and b*ALPL* variants than in patients with wt*ALPL* (Table 4). Fractures occurred in patients with densitometric osteopenia or osteoporosis, while they were not detected in patients with normal BMD, in all the three groups. In particular, among patients harboring p*ALPL* variants, four reported vertebral fractures (two osteoporotic and two osteopenic patients); among patients harboring b*ALPL* variants, five had fragility fractures (two osteoporotic and three osteopenic patients); in the wild-type *ALPL* group, one patient with osteopenia presented vertebral fractures. Logistic regression analysis showed that occurrence of vertebral fractures, considered as dependent variable, was not predicted by age, serum 25OHD and urine PEA levels, neck T-score, hypercalciuria/kidney stones and familiarity for fragility fractures, considered as independent variables.

**Table 3:** Anthropometric, biochemical, hormone and DXA-derived parameters: comparison between patients harboring pathogenic, benign variants and the wild-type alleles of the *ALPL* gene

Parameters	Pathogenic *ALPL* variants	Benign *ALPL* variants	Wild-type *ALPL* alleles	*P*
Age (years)	58.8 ± 3.3	59.1 ± 4.5	53.4 ± 3.8	0.535
BMI (kg/m^2^)	25.8 ± 0.9	21.7 ± 1.8	23.7 ± 2.1	0.453
Serum total ALP (U/L)	26.1 ± 2.3	32.3 ± 2.5	30.8 ± 2.3	0.274
Serum CTX (ng/mL)	0.541 ± 0.088	0.482 ± 0.081	0.285 ± 0.054	0.051
Serum total calcium (mg/dL)	9.2 ± 0.2	9.3 ± 0.1	9.4 ± 0.1	0.723
Serum phosphate (mg/dL)	3.8 ± 0.2	3.4 ± 0.2	3.3 ± 0.2	0.237
Serum 25OHD (ng/mL)	29.6 ± 5.2	39.0 ± 3.3	42.8 ± 3.8	0.112
Serum 25OHD < 30 ng/mL, *n* (%)	3 (60%)	1 (12.5%)	1 (11%)	0.080
Plasma PTH (pg/mL)	55.3 ± 8.0	41.2 ± 8.5	56.3 ± 7.8	0.205
UCa (mg/24 h)	186.4 ± 25.5	212.3 ± 21.8	154.4 ± 11.0	0.101
UCa (mg/kg/24 h)	2.7 ± 0.3	3.8 ± 0.5	2.6 ± 0.2	**0.048**
L1-L4 T-score	− 1.96 ± 0.74	− 1.94 ± 0.78	− 1.54 ± 0.37	0.849
Femoral neck T-score	− 2.14 ± 0.38	− 1.88 ± 0.27	− 1.50 ± 0.36	0.453
Total hip T-score	− 1.33 ± 0.59	− 1.41 ± 0.29	− 1.27 ± 0.30	0.950
DXA osteoporosis, *n* (%)	2 (40%)	4 (50%)	4 (44%)	0.937
Urinary PEA (mmol/mol creatinine)	5.8 ± 0.3	3.1 ± 0.7	2.3 ± 0.6	**0.005**
Serum PLP (µg/L)	30.5 ± 13.6	11.0 ± 0.9	10.6 ± 2.0	0.050

Data are presented as mean ± SEM and compared by oneway-ANOVA corrected by Holm-Sidak test for multiple comparisons. Categorical variables are presented as absolute and percentage frequencies and compared by Chi-square test

Bold values indicate statistically significant *P* values

*DXA* bone densitometric measurements, *BMI* body mass index, *ALP* alkaline phosphatase, *PEA* phosphoethanolamine, *PLP* Pyridoxalphosphate, *25OHD* 25-hydroxyvitamin D, *PTH* parathormone, *CTX* collagen type 1 cross-linked C-telopeptide, *UCa* urinary calcium excretion, *L1-L4* lumbar spine

**Table 4:** Occurrence of HPP-related symptoms and tamoxifen treatment: comparison between patients harboring the pathogenic/likely-pathogenic variants, the benign/likely benign variants and the wild-type alleles of *ALPL* gene

Risk factors	Pathogenic *ALPL* variants	Benign *ALPL* variants	Wild-type *ALPL* alleles	*P* value
Dental anomalies (%)	4/1 (80)*	3/5 (38)	0/9 (0)	**0.023**
Neurologic and musculoskeletal symptoms (%)	2/3 (40)	4/4 (50)§	0/9 (0)	**0.027**
Mineral metabolism anomalies (%)	1/4 (20)	5/3 (63)#	0/9 (0)	**0.014**
Familiarity for osteoporosis and/or fragility fractures (%)	3/2 (60)	5/3 (71)	1/8 (10)	0.061
All fractures (%)	4/1 (80)°°	5/3 (57)°	1/8 (20)	**0.022**
Vertebral fractures (%)	4/1 (80)	3/5 (37)	1/8 (20)	**0.037**
Increased urine PEA and/or serum PLP	5/0 (100)	6/2 (75)	5/4 (55)	0.199
Six-points score (mean ± SEM)	4.0 ± 0.6	3.4 ± 0.5	0.8 ± 0.2	**0.0001**
Tamoxifen treatment (%)	0/5 (0)^$$^	1/7 (13)^$^	6/3 (67)	**0.013**

Dental anomalies: recurrent caries (*n* = 3), early loss of permanent dental elements (*n* = 4), periodontal disease (*n* = 1); musculoskeletal and mineral metabolism symptoms: scoliosis (*n* = 1), dorsal hyperkiphosis (*n* = 2), osteoarthropathy and muscular pain (*n* = 4), seizure (*n* = 1); mineral metabolism anomalies: kidney stones (*n* = 3), hypercalciuria (*n* = 4); familiarity: first degree relatives with osteoporosis and/or fragility fractures; fractures: vertebral fractures (*n* = 8), ribs (*n* = 2), distal radius (*n* = 1), humerus (*n* = 1), clavicle (*n* = 1). Dicotomic variables are presented as the number of patients experiencing the indicated risk factor/number of patients free from the indicated risk factor; variables were compared by the Chi-Square test; 6-point scores are presented as mean ± SEM and they were analyzed by one-way ANOVA

Bold values indicate statistically significant *P* values

*P = 0.017 versus wild-type *ALPL* variants (WT)

^§^*P* = 0.029 versus WT

^#^*P* = 0.009 versus WT

°*P* = 0.049 versus WT

°°*P* = 0.023 versus WT

^$^*P* = 0.049 versus WT

^$$^*P* = 0.031 versus WT

Multiple risk factors common to osteoporosis and HPP, namely dental anomalies, neurologic and muscular-skeletal symptoms, mineral metabolism anomalies, familiarity for osteoporosis and/or fragility fractures have been investigated (Table 4). Dental anomalies and fractures, mainly vertebral fractures, were more frequently detected in patients harboring p*ALPL* compared with patients harboring wt*ALPL*; it should be noted that dental anomalies and fragility fractures were frequently detected also in patients harboring b*ALPL*. Musculoskeletal symptoms as well as mineral metabolism anomalies were more frequent in patients harboring b*ALPL* compared with patients harboring wt*ALPL* (Table 4).

Of note, looking at the group of hypophosphatasemic patients harboring wt*ALPL*, we found that seven patients experiencing low levels of serum ALP were treated with tamoxifen after diagnosis of hormone-sensitive breast cancer, referred for evaluation of CTIBL. All women were free of neoplasias; they were evaluated after at least 12 months from termination of chemio- and/or radiotherapy and treated with tamoxifen by 8–96 months. Serum ALP determinations, measured prior the beginning of the therapy with tamoxifen, were available only in two patients, and in one they were higher than 40 U/L.

### Biochemical features in hypophosphatasemic patients according the* ALPL* genotype

Mean serum total ALP levels were similar among the three groups, and they were not affected by hypovitaminosis D (34.0 ± 8.1 and 31.3 ± 1.7 U/L, in patients with serum 25OHD < 30 ng/mL versus patients with serum 25OHD > 30 ng/mL, respectively; *P* = 0.934 by Mann–Whitney test) (Table 3). Besides, serum CTX showed a trend to be higher in patients harboring p*ALPL* and b*ALPL* (Table 3). Mean serum total calcium, phosphate, and PTH levels were similar among the three groups, while urine calcium excretion levels tended to be higher in patients harboring b*ALPL* compared with levels detected in patients harboring p*ALPL* and wt*ALPL* (Table 3).

**Fig. 2:**
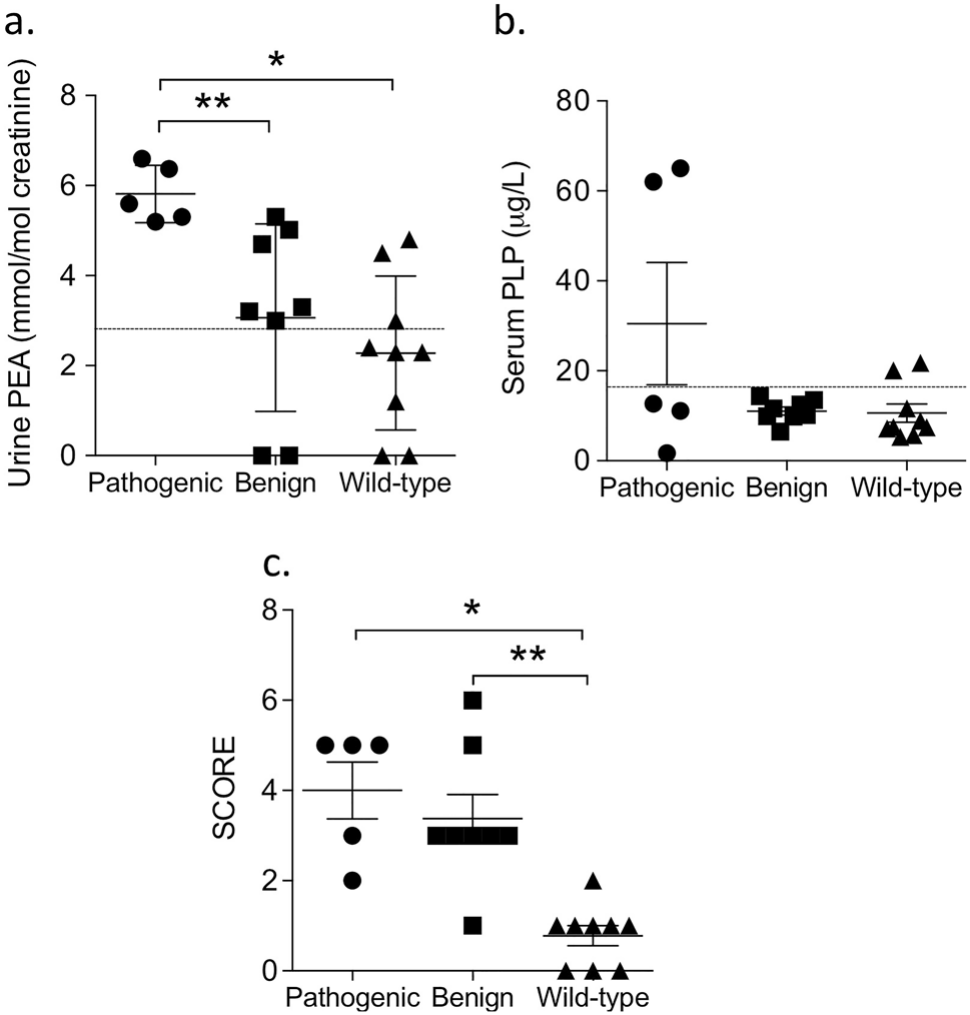
Differences among hypophosphatasemic patients harboring pathogenic/likely pathogenic *ALPL* variants, patients harboring benign/likely benign *ALPL* variants and the wild-type *ALPL* alleles. **a** Phosphetanolamine (PEA) concentrations in 24-h urine collections; *P = 0.017; **P = 0.008. **b** Serum pyridoxalphosphate (PLP) levels, *P* = 0.051. **c** Six-point scores; *P = 0.0004; **P = 0.0006. In panels a–b, data are presented as mean ± SEM and analyzed by oneway ANOVA; in panel c, data are presented as median, interquartile range, and analyzed by Kruskal–Wallis test

Considering the metabolites of alkaline phosphatase activity, mean urine PEA levels were significantly more elevated in patients harboring p*ALPL* than those detected in patients harboring b*ALPL* and wt*ALPL* (Table 3; Fig. 2a). By contrast, serum PLP levels were similar in the three groups, and in only two patients harboring p*ALPL*, PLP levels were consistently elevated (about threefold the upper limit of the normal range) (Table 3; Fig. 2b); indeed, p*ALPL* variants occurred in heterozygosis in all five patients, suggesting a genetic mild effect on ALP activity. The two metabolites PEA and/or PLP were altered not only in all patients harboring p*ALPL*, but also in three quarters of the patients harboring b*ALPL* and in about a half of those harboring wt*ALPL* (Table 4).

### The clinical and biochemical six-point score is predictive of ALPL gene aberrant variants

The six-point score distinguished patients harboring p*ALPL* and b*ALPL* from patients harboring wt*ALPL* (Fig. 2c). The six-point score was predictive of diagnosis of p*ALPL* with a *P* = 0.027 and an Odds Ratio of 1.99 (95% confidence interval 0.996, 3.984), while it was predictive of p*ALPL* or b*ALPL* with a *P* = 0.0001 and an Odds Ratio of 15.13 (95% confidence interval 1.497, 152.82).

## Discussion

The present study identified pathogenic variants of *ALPL* gene in one-fourth of hypophosphatasemic outpatients referred for diagnosis and management of osteopenia/osteoporosis, CTIBL and/or fragility fractures. In the present series, benign variants occurred in one-third of patients. This is the first study reporting about Italian hypophosphatasemic osteoporotic patients.

Previous series performing genetic analysis for the detection of *ALPL* aberrations in adult patients with persistent hypophosphatasemia at high risk for diagnosis of genetic HPP, defined as presence of clinical symptoms of adult HPP or positive family history, detected a prevalence of pathogenic variants of 43% [20] and 47% [21] or of rare/uncertain variants in 24% of patients [21]. Other studies evaluated the prevalence of HPP in hypophosphatasemic patients detected in different clinical settings. In a series of 50 hypophosphatasemic patients from US adult clinic population, pathogenic or likely-pathogenic or uncertain *ALPL* variants were detected in 84% of patients [22]. Besides, in a series of rheumatologic patients referred to the adult metabolic clinic of a single tertiary care center in Canada, including 24 individuals with persistently low serum levels of ALP, a prevalence of 58% of heterozygous pathogenic or likely pathogenic variants in *ALPL* was reported [23]. Lastly, a recent study investigated the prevalence of pathogenic *ALPL* variants in a series of 16 hypophosphatasemic patients identified from an UK osteoporosis clinic database, finding a prevalence of 87.5% [11]. The reported prevalence of p*ALPL* variants is discrepant with the lower prevalence detected in the present series of osteopenic/osteoporotic patients. Indeed, our series included a significant proportion of women with breast cancer treated with tamoxifen: excluding this subset of patients, that can be considered as affected by hypophophatasemia secondary to tamoxifen, the proportion of patients harboring p*ALPL* variants increased at 33%. Moreover, mean age of the hypophosphatasemic patients harboring *ALPL* variants included in the present series was 59 years (median 56 years). Though it was similar to the mean age reported in previous studies investigating general population [22], patients in the present series were elder than those from the rheumatologic series (median 49 years) [23] and those of the HPP registry (median 51.9 years) [24]. This finding suggests that among osteopenic/osteoporotic patients there are HPP patients with a mild symptomatic phenotype, which determines delay or failure in the diagnosis of HPP.

Typical clinical HPP complications were considered recurrent stress fractures, pseudofractures, osteomalacia, fracture healing disorders or dental abnormalities (including early loss of deciduous teeth before the age of 5 years with intact roots, extraction of several permanent teeth before the age of 50 years due to severe caries, narrow jaw or tooth loosening, malocclusion, severe periodontitis, atypical tooth morphology or visible dental hypomineralization); less specific symptoms of adult HPP included pyrophosphate arthropathy/pseudogout/chondrocalcinosis, low bone mineral density with T-Score ≤ − 2.5, musculoskeletal pain, weakness, calcifications, neurological/psychiatric symptoms such as frequent cephalgia, migraine or depression, and/or a positive family history of HPP. Evaluating the occurrence of these features in the present series of hypophosphatasemic adults in relation with the genetic background, we observed that in presence of similar conditions of bone demineralization, patients harboring p*ALPL* and b*ALPL* showed a higher prevalence of fragility fractures with respect to patients with wt*ALPL*. Indeed, fragility fractures were mostly vertebral fractures, which are not considered typical fractures occurring in HPP patients; though vertebral fractures are the most prevalent fractures in postmenopausal osteoporotic patients, in the present series they were not associated with the classic risk factors such as age, serum 25OHD levels, neck-T-score, familiarity for fragility fractures, and hypercalciuria/kidney stones. Similarly, dental anomalies were more frequent in patients harboring p*ALPL* and b*ALPL*. Unexpectedly, neurologic and musculoskeletal symptoms, though evaluated by patients’ complaints and not by objective scales, as well as mineral metabolism anomalies were more frequent in patients harboring b*ALPL*, showing a prevalence similar to that detected in a previously published series of adult HPP patients [24]. The overlap of clinical and biochemical features observed between patients with p*ALPL* and those with b*ALPL* raises doubts about the benign nature of the b*ALPL* variants. *ALPL* is active in bone, liver, kidney, and teeth. Though *ALPL* is expressed at high levels in the liver and kidney, its function in those organs remains elusive. Besides, the function of *ALPL* in non-mineralized tissues and the role of other substrates remain largely unresolved [25]. Alternatively, patients harboring b*ALPL* variants may display involvement of other genes affecting ALP activity promoting an HPP-like phenotype.

The ALP enzyme is required to metabolize vitamin B6 from a pyridoxal phosphate (PLP) substrate [25]; therefore, accumulation of the metabolites PLP and PEA is considered biochemical markers of reduced ALP activity. In line with the clinical findings, in the present series of patients, serum PLP levels did not significantly differ among the three groups, while mean urine PEA levels were higher in patients harboring p*ALPL* when compared with patients harboring b*ALPL* and wt*ALPL*. However, it is important to highlight that both serum PLP and urine PEA determinations were increased above the upper limit of the normal range also in three quarters of the patients harboring b*ALPL* and in about a half of those harboring wt*ALPL.* Considering the low prediction of HPP of serum PLP levels in the osteopenic/osteoporotic set of adult patients, the vitamin B6 challenge test may provide a more sensitive tool. It has been reported that an especially high serum PLP level after oral pyridoxine loading (i.e. 1/3 mg/kg body weight) marks carriers as well as patients with perinatal and infantile HPP [26]; however, the test should be preventively validated in an adult HPP cohort.

Last, the present study highlights the potential hypophosphatasemic effect of the tamoxifen treatment in women with hormone-sensitive breast cancer, suggesting the need for a careful evaluation of the ALP activity before starting the tamoxifen therapy in these women.

Admittedly, the present study suffers from some limitations, first of all the small size of the analyzed series, the retrospective design and the lack of serum ALP measurements before tamoxifen treatment in the women with hormone-sensitive breast cancer. However, patients were well characterized from the clinical, biochemical and genetic point of views.

In conclusion, detection of hypophosphatasemia in the management of osteopenic/osteoporotic adult patients represents a clinical challenge. Biochemical markers, such as PLP and PEA, are not able to clearly distinguish between patients harboring p*ALPL* and b*ALPL* from patients harboring wt*ALPL*. Considering clinical features and vitamin B6-related biomarkers may be more useful in predicting the detection of the aberrant *ALPL* variants in adult patients with osteopenia/osteoporosis and/or fragility fractures. Tamoxifen should be included among the hypophosphatasemic drugs.

## Data Availability

The datasets generated and analyzed during the current study are available at http://doi.org/10.5281/zenodo.4485746.
